# Use of a Nationwide Call Center for Ebola Response and Monitoring During a 3-Day House-to-House Campaign — Sierra Leone, September 2014

**Published:** 2015-01-16

**Authors:** Leigh Ann Miller, Thomas Sukalac, Emily Stanger, Reynold GB Senesi, Nick DeLuca, Patricia Dietz, Leslie Hausman, Peter H. Kilmarx, Jonathan Mermin

**Affiliations:** 1 Division of Global HIV/AIDS, Center for Global Health, CDC; 2 National Center for HIV/AIDS, Viral Hepatitis, STD, and TB Prevention, CDC; 3 Tony Blair Africa Governance Initiative; 4 Ministry of Public Health, Sierra Leone; 5 Division of HIV/AIDS, National Center for HIV/AIDS, Viral Hepatitis, STD, and TB Prevention, CDC; 6 Division of Foodborne, Waterborne, and Environmental Diseases, National Center for Emerging and Zoonotic Infectious Diseases, CDC

During May 23, 2014–January 10, 2015, Sierra Leone reported 7,777 confirmed cases of Ebola virus disease (Ebola) (1). In response to the epidemic, on August 5, Sierra Leone’s Emergency Operations Center established a toll-free, nationwide Ebola call center. The purpose of the call center is to encourage public reporting of possible Ebola cases and deaths to public health officials and to provide health education about Ebola to callers. This information also functions as an “alert” system for public health officials and supports surveillance efforts for the response. National call center dispatchers call district-level response teams composed of surveillance officers and burial teams to inform them of reported deaths and possible Ebola cases. Members of these response teams investigate cases and conduct follow-up actions such as transporting ill persons to Ebola treatment units or providing safe, dignified medical burials as resources permit. The call center continues to operate. This report describes calls received during a 3-day national campaign and reports the results of an assessment of the call center operation during the campaign.

The call center recorded all answered calls in a database. When the number of incoming calls exceeded the number of available lines, calls were not answered because there was no queue in which calls could be held for an available operator. Hence, unanswered calls were not recorded. The call center was staffed by 60 persons during two 12-hour shifts each day.

During September 19–21, the Sierra Leone government conducted a 3-day national campaign called “Ose-to-Ose Ebola Tok” (House-to-house Ebola talk), intended to provide education and galvanize support for the Ebola response. During the 3-day campaign, persons were required to stay in their homes, where they were visited by volunteer teams that provided Ebola education and sought to identify cases. More than 28,000 volunteers with knowledge of local resources and Ebola prevention information visited an estimated 75% of households nationwide during the 3-day campaign. Also, mass media and volunteers promoted using the call center to report possible cases of Ebola or to obtain more information.

An average of 1,100 calls per day was received during the 3-day campaign (Table); because of a computer malfunction on September 20, some data from that date were lost. Among the 3,299 callers during the 3-day period, 36% reported possible Ebola cases, 39% reported deaths, 9% asked for health information, 2% asked questions related to quarantine, and 23% reported other issues (e.g., questions or concerns regarding the campaign). More than one call could have reported the same death or possible case. During the campaign, 47% of reported calls came from the Western Urban and 15% came from the Western Rural district. Compared with day 1, on day 3 total call volume was 10% higher, and the number of calls reporting possible Ebola cases was 28% higher. The number of calls reporting deaths was 14% lower.

Each day during the campaign, call center dispatchers telephoned district-level response teams to notify them of reported deaths and possible cases. To determine whether calls received resulted in action by a district-level response team, the call center staff conducted a follow-up survey 1 week after the campaign. During September 26–27, the call center telephoned 191 households in Bombali, Port Loko, Western Urban, and Western Rural districts that had reported deaths (96) and possible cases (95) during September 19–21. The districts were selected by convenience and call center dispatchers recorded the number of days between the call and the response (i.e., when a burial or surveillance team visited the home).

From these four districts, among households that had reported a death, 44% reported receiving a response the same day; 37% reported a response the next day; 7% reported a response within 2–3 days of calling; and 12% reported receiving no response by a district team. Among households that reported possible cases, 31% reported receiving a response the same day; 14% reported a response the next day; 6% reported a response within 2–3 days of calling, and 50% reported there was no response from district teams.

The findings in this report are subject to at least three limitations. First, a computer malfunction resulted in incomplete data for September 20. Second, the data are not generalizable to other areas. Finally, the usefulness of call center data was limited in trying to understand why some district team responses were delayed or incomplete.

Sierra Leone’s 3-day national campaign was a highly publicized effort to raise Ebola awareness and educate the public about prevention, home care, and treatment options. The call center was used to answer questions from citizens and helped the government manage the outbreak response. In the follow-up survey, a response on the same or next day was received for 81% of reported deaths but only 45% of possible cases. Because treatment and isolation of possible cases are essential to control the epidemic, this finding suggested an urgent need to scale-up response services. Since October, there have been increases in Ebola treatment units, burial teams, and coordinated call center response at the district level that have helped to improve response capacity. Call centers can be used to improve allocation of resources, provide the public with a credible source for assistance and information, monitor programs, and possibly to assist in decreasing rates of local transmission by facilitating prompt transfer of ill persons to hospitals or Ebola treatment units and providing prompt and safe burial of persons who have died in their homes.

## Figures and Tables

**TABLE:** Number of incoming calls, reported deaths, and reported possible Ebola patients, by district — nationwide Ebola call center, Sierra Leone, September 19–21, 2014

District	September 19	September 20*	September 21	Total
**No. of incoming calls**				
Bombali	73	52	76	201
Port Loko	96	44	101	241
Western Rural	166	125	190	481
Western Urban	503	389	663	1,555
11 other districts†	355	188	278	821
**Total**	**1,193**	**798**	**1,308**	**3,299**
**No. of reported deaths**				
Bombali	17	6	14	37
Port Loko	14	7	31	52
Western Rural	96	76	81	253
Western Urban	220	203	250	673
11 other districts†	163	53	65	281
**Total**	**510**	**345**	**441**	**1,296**
**No. of reported possible Ebola cases**				
Bombali	34	26	39	99
Port Loko	37	21	39	97
Western Rural	37	31	63	131
Western Urban	178	126	284	588
11 other districts†	125	62	100	287
**Total**	**411**	**266**	**525**	**1,202**

* Data for September 20 are incomplete because of a computer malfunction resulting in data loss.

† The 11 districts were Bo, Bonthe, Bonthe Island, Kailahun, Kambia, Kenema, Koinadugu, Moyamba, Pujehun, and Tonkolili.
